# Cudratricusxanthone A Inhibits Lipid Accumulation and Expression of Inducible Nitric Oxide Synthase in 3T3-L1 Preadipocytes

**DOI:** 10.3390/ijms22020505

**Published:** 2021-01-06

**Authors:** Hyo-Shin Kwon, Gil-Saeng Jeong, Byeong-Churl Jang

**Affiliations:** 1 Department of Molecular Medicine, College of Medicine, Keimyung University, Daegu 42601, Korea; 1112814@stu.kmu.ac.kr; 2 College of Pharmacy, Keimyung University, Daegu 42601, Korea; gsjeong@kmu.ac.kr

**Keywords:** Cudratricusxanthone A, 3T3-L1, C/EBP-α, PPAR-γ, AMPK, iNOS

## Abstract

Cudratricusxanthone A (CTXA) is a natural bioactive compound extracted from the roots of *Cudrania tricuspidata* Bureau and has been shown to possess anti-inflammatory, anti-proliferative, and hepatoprotective activities. However, at present, anti-adipogenic and anti-inflammatory effects of CTXA on adipocytes remain unclear. In this study, we investigated the effects of CTXA on lipid accumulation and expression of inducible nitric oxide synthase (iNOS) and cyclooxygenase (COX)-2, two known inflammatory enzymes, in 3T3-L1 preadipocytes. Strikingly, CTXA at 10 µM markedly inhibited lipid accumulation and reduced triglyceride (TG) content during 3T3-L1 preadipocyte differentiation with no cytotoxicity. On mechanistic levels, CTXA at 10 µM suppressed not only expression levels of CCAAT/enhancer-binding protein-α (C/EBP-α), peroxisome proliferator-activated receptor-γ (PPAR-γ), fatty acid synthase (FAS), and perilipin A, but also phosphorylation levels of signal transducer and activator of transcription-3 (STAT-3) and STAT-5 during 3T3-L1 preadipocyte differentiation. In addition, CTXA at 10 µM up-regulated phosphorylation levels of cAMP-activated protein kinase (AMPK) while down-regulating expression and phosphorylation levels of acetyl-CoA carboxylase (ACC) during 3T3-L1 preadipocyte differentiation. Moreover, CTXA at 10 µM greatly attenuated tumor necrosis factor (TNF)-α-induced expression of iNOS, but not COX-2, in 3T3-L1 preadipocytes. These results collectively demonstrate that CTXA has strong anti-adipogenic and anti-inflammatory effects on 3T3-L1 cells through control of the expression and phosphorylation levels of C/EBP-α, PPAR-γ, FAS, ACC, perilipin A, STAT-3/5, AMPK, and iNOS.

## 1. Introduction

Obesity is a major health concern often deteriorating life expectancy and increasing risks of many human diseases, such as type 2 diabetes mellitus, cardiovascular diseases, hypertension, non-alcoholic fatty liver disease, osteoarthritis, and cancer [1]. The alarming fact that the incidence of obesity has elevated steadily in the last decades, and almost half of the world’s population is obese or overweight [2] has prompted the need for identifying novel cost-effective interventions that are capable of controlling obesity with minimal side effects. Given that obesity is defined as an increase in body mass fat resulting from excessive preadipocyte differentiation in the human body [3,4], and also as a low or systemic chronic inflammation [5], any substance that inhibits excessive preadipocyte differentiation and inflammatory responses in (pre)adipocytes can be therefore considered as a potential anti-obesity agent. 

Preadipocyte differentiation, also called adipogenesis, is a multi-step process that occurs in the form of cellular, morphological, genetic, and biochemical changes. Through this process, fibroblast-like preadipocytes are converted into mature adipocytes that are filled with lipid droplets (LDs) [6,7,8]. A wealth of information indicates that many intracellular proteins participate in preadipocyte differentiation. Indeed, studies have demonstrated that several adipogenic transcription factors, including the family of CCAAT/enhancer-binding proteins (C/EBPs), peroxisome proliferator-activated receptors (PPARs), and signal transducer and activator of transcription (STAT) proteins play a pivotal role in preadipocyte differentiation by regulating the expression of genes related to this process [9,10]. It also has been shown that fatty acid synthase (FAS), acetyl-CoA carboxylase (ACC), and perilipin A, which regulate lipogenesis and LDs maturation/stabilization are crucial for preadipocyte differentiation [11,12,13]. Moreover, there is evidence pointing out the positive role of numerous signaling protein kinases, such as cAMP-activated protein kinase (AMPK), protein kinase A (PKA), extracellular signal-regulated protein kinase-1/2 (ERK-1/2), p38 mitogen-activated protein kinase (MAPK), and protein kinase C (PKC) in preadipocyte differentiation [14,15,16,17]. 

Mounting evidence illustrates that (pre)adipocytes in the adipose tissues (ATs) are exposed to different endogenic and exogenic stimuli, such as pro-inflammatory cytokines, free fatty acids (FFAs), or lipopolysaccharide (LPS) [18,19,20]. Tumor necrosis factor-α (TNF-α) is a pro-inflammatory cytokine that stimulates (pre)adipocytes to express and secrete many inflammatory mediators and chemokines [21], thereby exacerbating inflammation and recruiting macrophages in the ATs [22]. Cyclooxygenase-2 (COX-2) and inducible nitric oxide synthase (iNOS) are known inflammatory enzymes that are involved in the production of prostaglandins (PGs) and NO, respectively [23,24]. It has notably been demonstrated that TNF-α is a strong inducer of COX-2 and iNOS in many types of cells, including (pre)adipocytes [25]. These results suggest inhibition (or inhibitor) of TNF-α-induced COX-2 and iNOS expression in (pre)adipocytes as a potential target in alleviating obesity inflammation. 

Cudratricusxanthone A (CTXA) is a bioactive component isolated from the roots of *Cudrania tricuspidata* Bureau and has anti-inflammation, anti-cancer, anti-osteoclast differentiation, neuroprotection, and anti-thrombotic effects [26,27,28,29,30]. However, as of now, the anti-obesity effect of CTXA on adipocytes is unknown. In this study, we investigated the regulatory effects of CTXA on lipid accumulation and TNF-α-induced expression of COX-2 and iNOS in 3T3-L1 cells, a murine white (pre)adipocyte. Here we report firstly that CTXA at 10 µM has strong anti-adipogenic and anti-inflammatory effects in 3T3-L1 cells, and these effects are mediated through modulation of the expression and phosphorylation levels of C/EBP-α, PPAR-γ, FAS, ACC, perilipin A, STAT-3/5, AMPK, and iNOS.

## 2. Results

### 2.1. CTXA Induces a Concentration-Dependent Inhibition of Lipid Accumulation during 3T3-L1 Preadipocyte Differentiation

**Figure 1:**
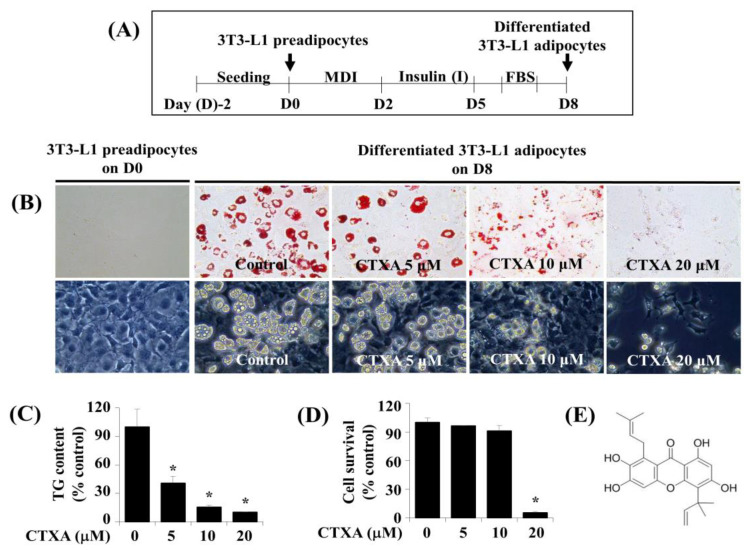
Effects of Cudratricusxanthone A (CTXA) on lipid accumulation, triglyceride (TG) content, and cell survival during 3T3-L1 preadipocyte differentiation. (**A**) The experimental protocol for 3T3-L1 preadipocyte differentiation. (**B**) Measurement of lipid accumulation in 3T3-L1 preadipocytes (D0) and adipocytes (D8) grown in the absence or presence of CTXA at the designated concentrations by Oil Red O staining (Upper panel) or microscopic observation (Lower panel). (**C**) Measurement of TG content in 3T3-L1 adipocytes (D8) that were grown in the absence or presence of CTXA at the designated concentrations by Adipo-red assay. Each experiment was conducted independently in triplication. Data are mean ± SE of three independent experiments. * *p* < 0.05 compared to the value of vehicle control at the indicated time. (**D**) Measurement of the number of live cells in 3T3-L1 adipocytes (D8) that were grown in the absence or presence of CTXA at the designated concentrations by cell survival analysis. Each experiment was conducted independently in triplication. Data are mean ± SE of three independent experiments. * *p* < 0.05 compared to the value of vehicle control at the indicated time. (**E**) The chemical structure of CTXA.

Figure 1A is the experimental protocol of 3T3-L1 preadipocyte differentiation used in this study. Initially, we investigated the effect of different concentrations of CTXA on cellular lipid accumulation during the differentiation of 3T3-L1 preadipocytes into adipocytes using Oil Red O staining. Of note, as shown in Figure 1B (Upper panels), CTXA concentration-dependently suppressed lipid accumulation in 3T3-L1 cells on D8 of differentiation. The CTXA’s lipid-lowering effect on D8 of 3T3-L1 preadipocyte differentiation was also confirmed by microscopic observation (Low panels). Given that cellular lipids are mainly stored in the form of triglyceride (TG) in differentiated adipocytes [31], we next analyzed the effect of different concentrations of CTXA on cellular TG content on D8 of 3T3-L1 preadipocyte differentiation using an Adipo-red assay. As shown in Figure 1C, there was a dose-dependent reduction of cellular TG content in 3T3-L1 cells on D8 of differentiation. Next, we examined whether CTXA at the doses tested affects growth (survival) of 3T3-L1 cells on D8 of 3T3-L1 preadipocyte differentiation using cell count analysis. As shown in Figure 1D, while CTXA at 5 or 10 µM did not affect survival of 3T3-L1 cells, CTXA at 20 µM reduced these cells’ survival by approximately 95%. Thus, the maximal lipid-lowering effect by CTXA at 20 µM on D8 of 3T3-L1 preadipocyte differentiation seemed to be attributable to its cytotoxicity. The chemical structure of CTXA is depicted in Figure 1E. Because of the strong lipid-lowering effect with no cytotoxicity on D8 of 3T3-L1 preadipocyte differentiation, we selected the 10 μM concentration of CTXA in further studies. 

### 2.2. CTXA at 10 µM Reduces Protein Expression Level of C/EBP-α and PPAR-γ as Well as Phosphorylation Level of STAT-3 and STAT-5 during 3T3-L1 Preadipocyte Differentiation

**Figure 2:**
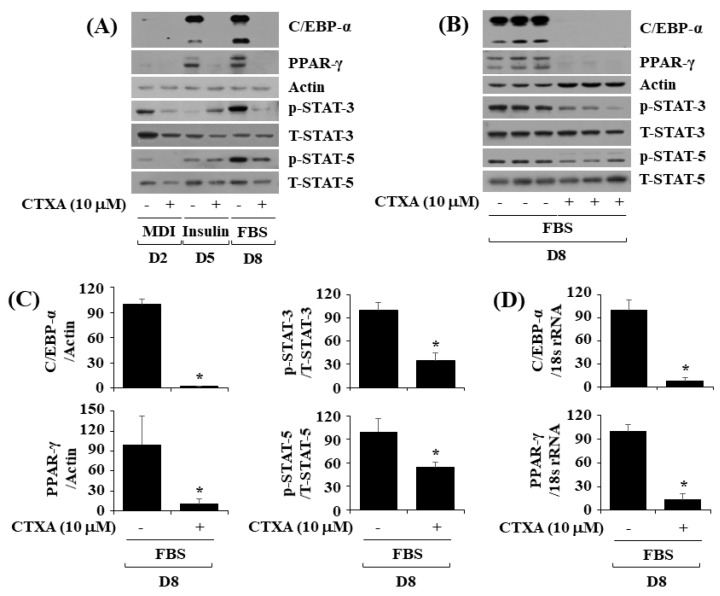
Effects of Cudratricusxanthone A (CTXA) on expression and phosphorylation level of CCAAT/enhancer-binding protein-α (C/EBP-α), peroxisome proliferator-activated receptor-γ (PPAR-γ), signal transducer and activator of transcription-3 (STAT-3), and STAT-5 during 3T3-L1 preadipocyte differentiation. (**A**) 3T3-L1 preadipocytes were differentiated with induction medium containing MDI, insulin, and fetal bovine serum (FBS) in the absence (control; 0.1 % DMSO) or presence of CTXA (10 µM), and harvested at day 2 (D2), D5, and D8, respectively. At each time point, whole cell lysates were prepared and analyzed by Western blotting with respective antibodies. *p*-STAT-3, phosphorylated STAT-3; T-STAT-3, total STAT-3; *p*-STAT-5, phosphorylated STAT-5; T-STAT-5, total STAT-5. (**B**) Western blotting with respective antibodies in triplicate experiments on D8. (**C**) Densitometry analysis of (**B**). * *p* < 0.05 compared to vehicle control. (**D**) 3T3-L1 preadipocytes were differentiated with induction medium containing MDI, insulin, and FBS in the absence (control; 0.1 % DMSO) or presence of CTXA (10 µM), and harvested at D8. Total cellular RNA was extracted and analyzed by real-time qPCR with respective primers. Data are mean ± SE of three independent experiments. * *p* < 0.05 vs. control.

Next, we analyzed whether CTXA at 10 µM interferes with expression and phosphorylation of C/EBP-α, PPAR-γ, STAT-3, and STAT-5, key adipogenic transcription factors, during 3T3-L1 preadipocyte differentiation using Western blotting. As depicted in Figure 2A, CTXA at 10 µM almost completely inhibited expression of C/EBP-α and PPAR-γ at the protein level on D5 and D8 of 3T3-L1 preadipocyte differentiation. Moreover, CTXA at 10 µM could largely inhibit phosphorylation of STAT-3 and STAT-5 on D2 and D8 of 3T3-L1 preadipocyte differentiation. Triplicate experiments, as shown in Figure 2B, further demonstrated the ability of CTXA at 10 µM to significantly inhibit not only expression of C/EBP-α and PPAR-γ but also phosphorylation of STAT-3 and STAT-5 on D8 of 3T3-L1 preadipocyte differentiation. Densitometric data of Figure 2B for the expression level of C/EBP-α or PPAR-γ normalized to that of control actin, and phosphorylation level of STAT-3 or STAT-5 normalized to the expression level of total STAT-3 or STAT-5 on D8 of 3T3-L1 preadipocyte differentiation are shown in Figure 2C. As further shown in Figure 2D, data of real-time qPCR analysis from triplicate experiments for measurement of mRNA expression level of C/EBP-α or PPAR-γ normalized to that of control 18S rRNA revealed that CTXA at 10 µM significantly reduced transcripts of C/EBP-α and PPAR-γ on D8 of 3T3-L1 preadipocyte differentiation.

### 2.3. CTXA at 10 µM Downregulates Expression Level of FAS and Perilipin A during 3T3-L1 Preadipocyte Differentiation

**Figure 3:**
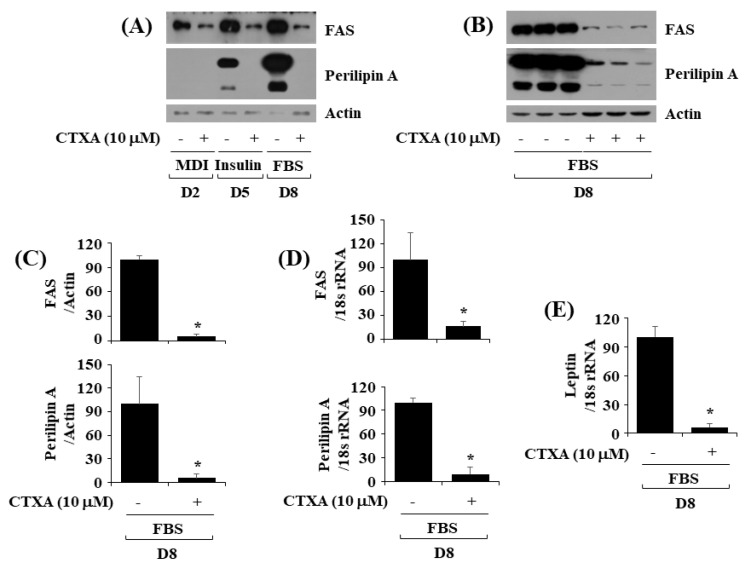
Effects of CTXA on expression level of perilipin A and fatty acid synthase (FAS) during 3T3-L1 preadipocyte differentiation. (**A**) 3T3-L1 preadipocytes were differentiated with induction medium containing MDI, insulin, and FBS in the absence (control; 0.1 % DMSO) or presence of CTXA (10 µM), and harvested at day 2 (D2), D5, and D8, respectively. At each time point, whole cell lysates were prepared and analyzed by immunoblot analysis with respective antibodies. (**B**) Western blot analysis in triplicate experiments on D8. (**C**) Densitometry analysis of (**B**). * *p* < 0.05 compared to control. (**D**,**E**) Total cellular RNA was extracted at D8 and analyzed by real-time qPCR with respective primers. Data are mean ± SE of three independent experiments. * *p* < 0.05 vs. control.

Next, we investigated the effect of CTXA at 10 µM on expression of FAS, an enzyme responsible for fatty acid synthesis [11,32] and perilipin A, a lipid droplet-binding and stabilizing protein [13,33], during 3T3-L1 preadipocyte differentiation. As shown in Figure 3A, CTXA at 10 μM strongly inhibited FAS protein expression on D2, D5, and D8 of 3T3-L1 preadipocyte differentiation. CTXA at 10 μM also greatly blocked perilipin A protein expression on D5 and D8 of 3T3-L1 preadipocyte differentiation. Results of Western blotting from triplicate experiments, as shown in Figure 3B, also revealed the ability of CTXA at 10 µM to largely inhibit expression of FAS and perilipin A proteins on D8 of 3T3-L1 preadipocyte differentiation. Densitometric data of Figure 3B for protein expression level of FAS or perilipin A normalized to that of control actin on D8 of 3T3-L1 preadipocyte differentiation are shown in Figure 3C. As further shown in Figure 3D, results of real-time qPCR from triplicate experiments for mRNA expression level of FAS or perilipin A normalized to that of control 18S rRNA demonstrated that CTXA at 10 µM significantly reduced transcripts of FAS or perilipin A on D8 of 3T3-L1 preadipocyte differentiation. In addition, as shown in Figure 3E, CTXA at 10 µM could significantly inhibit leptin mRNA expression on D8 of 3T3-L1 preadipocyte differentiation. 

### 2.4. CTXA at 10 µM Alters Phosphorylation and Expression Level of AMPKα, LKB1, and ACC during 3T3-L1 Preadipocyte Differentiation

**Figure 4:**
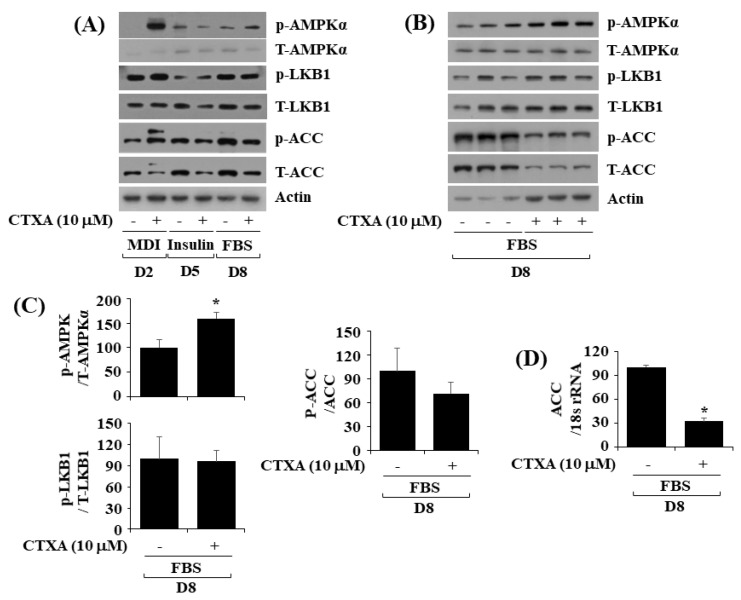
Effects of CTXA on expression and phosphorylation level of cAMP-activated protein kinase (AMPK)α, liver kinase B1 (LKB1), and acetyl-CoA carboxylase (ACC) during 3T3-L1 preadipocyte differentiation. (**A**) 3T3-L1 preadipocytes were differentiated with induction medium containing MDI, insulin, and FBS in the absence (control; 0.1 % DMSO) or presence of CTXA (10 µM), and harvested at day 2 (D2), D5, and D8, respectively. At each time point, whole cell lysates were prepared and analyzed by immunoblot analysis with respective antibodies. *p*-AMPKα, phosphorylated AMPKα; T- AMPKα, total AMPKα; *p*-LKB1, phosphorylated LKB1; T-LKB1, total LKB1; *p*-ACC, phosphorylated ACC; T-ACC, total ACC. (**B**) Western blot analysis in triplicate experiments on D8. (**C**) Densitometry analysis of (**B**). * *p* < 0.05 compared to vehicle control. (**D**) Total cellular RNA was extracted at D8 and analyzed by real-time qPCR with respective primers. Data are mean ± SE of three independent experiments. * *p* < 0.05 vs. control.

AMPK is a heterotrimeric protein complex that is composed of α, β, and γ subunits [34], and plays a key role in cellular energy homeostasis [35]. There is much evidence that activation of AMPK inhibits lipid accumulation (adipogenesis) in 3T3-L1 preadipocyte differentiation [36]. The α subunit of AMPK (AMPKα) contains its catalytic domain where AMPK becomes activated when phosphorylation takes place at T172 [37,38,39]. This promptly led us to investigate the effect of CTXA at 10 µM on phosphorylation and expression level of AMPKα during 3T3-L1 preadipocyte differentiation. Of interest, as depicted in Figure 4A, CTXA at 10 µM elevated phosphorylation (T172) of AMPKα on D2 and D8 of 3T3-L1 preadipocyte differentiation. In addition, CTXA at 10 µM could increase phosphorylation (S79) of ACC, a downstream effector of AMPK [38], on D2 of 3T3-L1 preadipocyte differentiation. Of further note, CTXA at 10 µM also largely reduced expression of ACC on D5 and D8 of 3T3-L1 preadipocyte differentiation. CTXA at 10 µM had no or little effect on phosphorylation (S428) and expression level of liver kinase B1 (LKB1), an upstream kinase of AMPKα [39], in 3T3-L1 preadipocyte differentiation at times applied. Results of Western blotting from triplicate experiments, as shown in Figure 4B, further revealed the ability of CTXA at 10 µM to elevate phosphorylation of AMPKα but decrease in phosphorylation and expression of ACC on D8 of 3T3-L1 preadipocyte differentiation. Densitometric data of Figure 4B for protein phosphorylation level of AMPKα, LKB1 and ACC normalized to expression level of total AMPKα, LKB1, and ACC on D8 of 3T3-L1 preadipocyte differentiation are shown in Figure 4C, respectively. As further shown in Figure 4D, results of real-time qPCR from triplicate experiments for mRNA expression level of ACC normalized to that of control 18S rRNA displayed that CTXA at 10 µM significantly down-regulated mRNA expression of ACC on D8 of 3T3-L1 preadipocyte differentiation. 

### 2.5. CTXA at 10 µM Does Not Stimulate Glycerol Release and Phosphorylation of HSL in Mature 3T3-L1 Adipocytes

**Figure 5:**
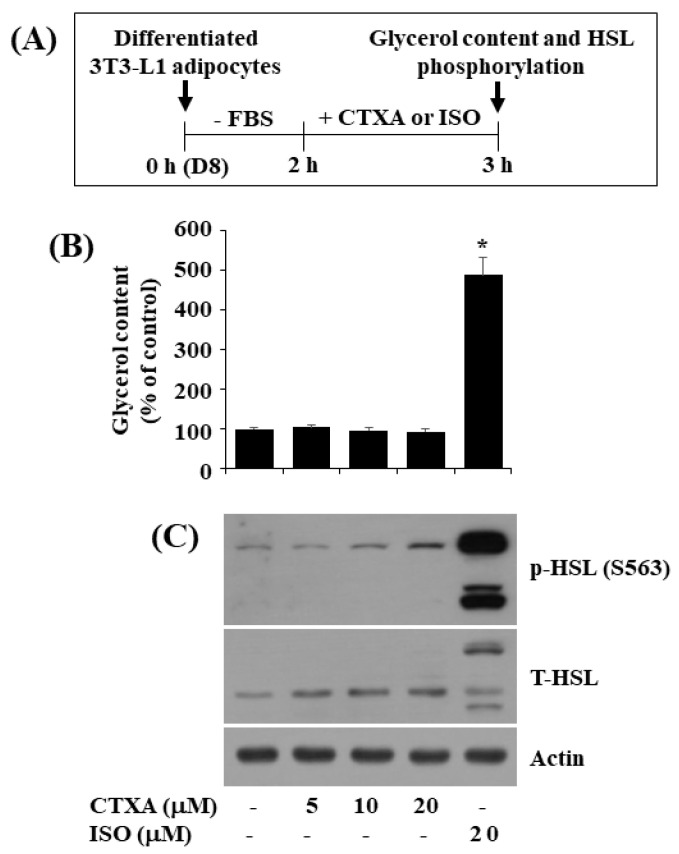
Effects of CTXA on glycerol release and expression and phosphorylation level of hormone-sensitive lipase (HSL) in differentiated 3T3-L1 cells. (**A**) The experimental scheme for measurement of glycerol content and HSL phosphorylation in differentiated 3T3-L1 cells. (**B**) Differentiated 3T3-L1 cells on D8 (0 h) were serum-starved for 2 h and then grown in the absence (control; 0.1 % DMSO) or presence of CTXA (5, 10, and 20 µM) or isoproterenol (ISO) (10 µM) for an additional 3 h. Glycerol content in culture medium from control or drug (CTXA or ISO)-treated cells was analyzed in triplicate. Data are mean ± SE of three independent experiments. * *p* < 0.05 vs. control. (**C**) After the treatment mentioned above in (**A**), whole cell lysates were prepared and analyzed by Western blotting with respective antibodies. *p*-HSL, phosphorylated HSL; T-HSL, total HSL.

Next, we examined the effect of CTXA at 10 µM on lipolysis in differentiated (mature) 3T3-L1 cells. In this study, the CTXA’s lipolysis-inducing effect was assessed by its ability to elevate glycerol release and hormone-sensitive lipase (HSL) phosphorylation (S563), and isoproterenol (ISO), a lipolysis-inducing agent [40], was used as a positive control. The experimental protocol for assessment of glycerol release and HSL phosphorylation is depicted in Figure 5A. As expected, ISO at 20 μM for 3 h markedly elevated glycerol content in the culture media of differentiated 3T3-L1 cells (Figure 5B). However, CTXA at 5 to 20 μM for 3 h did not elevate glycerol content in these cells. Furthermore, while ISO at 20 μM for 3 h strongly increased HSL phosphorylation in differentiated 3T3-L1 cells, CTXA at 10 μM for 3 h had no or little effect on it (Figure 5C). Expression level of control actin and total HSL remained unchanged under these experimental conditions.

### 2.6. CTXA at 10 µM Inhibits TNF-α-Induced Expression of iNOS, but Not COX-2, in 3T3-L1 Preadipocytes

**Figure 6:**
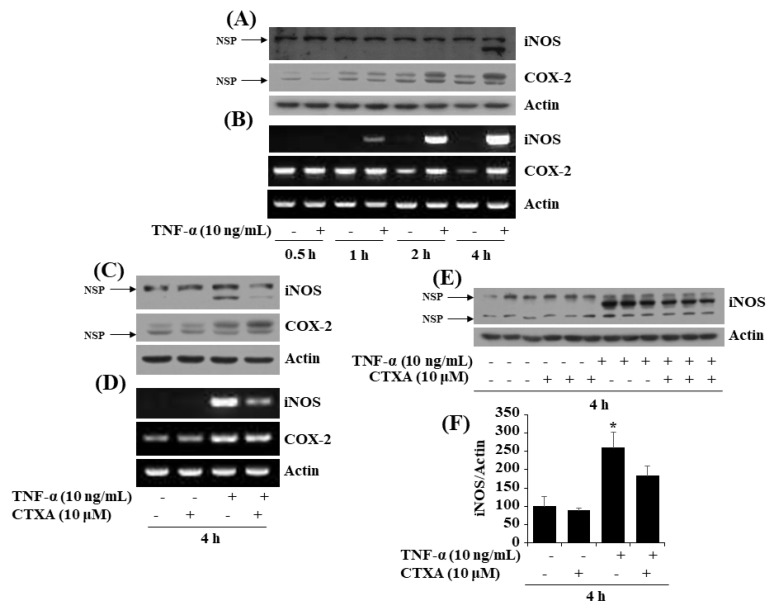
Effects of CTXA on tumor necrosis factor (TNF)-α-induced expression level of inducible nitric oxide synthase (iNOS) and cyclooxygenase-2 (COX-2) in 3T3-L1 preadipocytes. (**A**,**B**) 3T3-L1 preadipocytes were treated with or without TNF-α (10 ng/mL) for designated periods. At each time point, whole cell lysate or total RNA was isolated and analyzed for iNOS and COX-2 by Western blotting or RT-PCR. (**C**,**D**) 3T3-L1 preadipocytes were treated with or without CTXA (10 µM) in the presence or absence of TNF-α (10 ng/mL) for 4 h. Whole cell lysate or total RNA was isolated and analyzed for iNOS and COX-2 by Western blotting or RT-PCR. (**E**) After the treatment mentioned above in (**C**,**D**), whole cell lysates were prepared from three independent experiments and analyzed by Western blotting with respective antibodies. (**F**) Densitometry analysis of (**E**). * *p* < 0.05 compared to vehicle control. NSP, non-specific protein.

To see any anti-inflammatory effect, we next investigated whether TNF-α at 10 ng/mL induces expression of COX-2 and iNOS in 3T3-L1 preadipocytes over time, and treatment with CTXA at 10 μM could interfere with it. As shown in Figure 6A,B, treatment with TNF-α at 10 ng/mL for 4 h maximally induced expression of COX-2 and iNOS at both protein and mRNA levels in 3T3-L1 preadipocytes, respectively. Of note, CTXA treatment at 10 µM for 4 h greatly suppressed TNF-α-induced protein and mRNA expression of iNOS, but not COX-2, in 3T3-L1 preadipocytes (Figure 6C,D). Furthermore, results of Western blotting in triplicate experiments, as shown in Figure 6E, confirmed the ability of CTXA at 10 µM to block TNF-α-induced iNOS protein expression in 3T3-L1 preadipocytes. Densitometric data of Figure 6E for protein expression level of iNOS normalized to that of control actin in 3T3-L1 preadipocytes treated with TNF-α in the absence or presence of CTXA at 10 µM for 4 h are shown in Figure 6F. It should be noted that there are non-specific proteins with a same or similar epitope (some amino acid sequences) recognized by the iNOS or COX-2 antibody used herein, which are indicated in Figure 6A,C, and/or 6E with arrows labeled with NSP (non-specific protein). This notion is based on the fact that expression of NSP is not inducible by TNF-α treatment at the times tested, given that iNOS and COX-2 are inducible enzymes whose expressions are rapidly inducible by extracellular stimuli including TNF-α herein.

## 3. Discussion

Although there is much evidence addressing that CTXA has anti-inflammatory, anti-cancerous, and anti-osteoclast differentiation activities [27,28,29], as of now, this natural substance’s anti-obesity effect remains unclear. In this study, we report firstly that CTXA at 10 µM has strong anti-adipogenic and anti-inflammatory effects on 3T3-L1 preadipocytes, and these effects are mediated through modulation of the expression and phosphorylation level of C/EBP-α, PPAR-γ, FAS, ACC, perilipin A, STAT-3/5, AMPK, and iNOS.

Through initial experiments, we have shown that CTXA at 10 µM greatly inhibits lipid accumulation and reduces TG content during the differentiation of 3T3-L1 preadipocyte into adipocytes, pointing out its anti-adipogenic (lipid-lowering) effect. As mentioned before, the differentiation process requires fibroblast-like preadipocytes to develop into lipid-laden mature (differentiated) adipocytes [6,7,8,41], and numerous adipogenic transcription factors, including CCAAT/enhancer-binding proteins (C/EBPs), peroxisome proliferator-activated receptors (PPARs), and signal transducer and activator of transcription (STAT) proteins participate in the process [9,10,42,43,44]. As of now, little is known about CTXA regulation of C/EBP-α, PPAR-γ, and STAT-3 in the preadipocyte differentiation process. Strikingly, we herein have shown that CTXA at 10 µM strongly downregulates C/EBP-α and PPAR-γ at both protein and mRNA levels in 3T3-L1 preadipocyte differentiation, which may further point out that CTXA-mediated C/EBP-α and PPAR-γ down-regulation is due to their transcriptional repression. Moreover, the present study has demonstrated the ability of CTXA at 10 µM to largely interfere with phosphorylation of STAT-3 and STAT-5 without altering their total protein level in 3T3-L1 preadipocyte differentiation. These results collectively suggest that CTXA’s anti-adipogenic effect on 3T3-L1 cells is closely linked to the reduced expression and phosphorylation levels of C/EBP-α, PPAR-γ, and STAT-3/5. 

It is known that the process of preadipocyte differentiation is accompanied by the synthesis of fatty acid, also called lipogenesis and stabilization of LDs [4,7,41]. FAS and ACC are important lipogenic enzymes involved in the synthesis of fatty acid [11,32]. Perilipin A is a protein that binds to and stabilizes cellular LDs, which thereby plays a crucial role in lipid accumulation or storage in preadipocyte differentiation process [13,33,45,46]. To date, CTXA regulation of FAS, ACC, and perilipin A in this preadipocyte differentiation process is unknown. Of note, we herein have shown that CTXA at 10 µM greatly lowers expression of FAS and perilipin A at their protein and mRNA level during 3T3-L1 preadipocyte differentiation at times tested (D2, D5, and/or D8). In the case of ACC, however, CTXA treatment at 10 µM substantially increases level of phosphorylated ACC, which are inactive forms of ACC [38], at the early (D2) stage of 3T3-L1 preadipocyte differentiation, while it largely reduces the level of total ACC at the middle (D5) and late (D8) stage of the cell differentiation. These results suggest that CTXA’s anti-adipogenic/anti-lipogenic (lipid-lowering) effects are further attributable to the reduced expression of FAS, ACC, and perilipin A. 

AMPK is a metabolic protein that plays a pivotal role in the regulation of cellular energy homeostasis [14]. Until now, CXTA regulation of AMPK in the preadipocyte differentiation process has been unknown. Of interest, we herein have found that CTXA at 10 µM induces high AMPK phosphorylation on T172, which is an active form of AMPK [47,48], at the early (D2) and late (D8) stage of 3T3-L1 preadipocyte differentiation. Given that AMPK is an upstream regulator of ACC phosphorylation [38], and CTXA at 10 µM also induces ACC phosphorylation on D2 of 3T3-L1 preadipocyte differentiation herein, it is, therefore, conceivable that CTXA induces activation of AMPK, which is responsible for ACC phosphorylation at the early (D2) stage of 3T3-L1 preadipocyte differentiation. Further assuming that activation of AMPK inhibits preadipocyte differentiation [37,40,47,48,49], it is likely that activation of AMPK may further contribute to CTXA’s anti-adipogenic and anti-lipogenic effects. It has been previously shown that phosphorylation of AMPK is induced by several upstream kinases including LKB1 [39]. However, in the current study, CTXA at 10 µM is unable to induce LBK1 phosphorylation during 3T3-L1 preadipocyte differentiation process. These results address that CTXA-induced AMPK phosphorylation during 3T3-L1 preadipocyte differentiation herein is not through LKB1 but other upstream kinases and/or mechanisms. Given that AMPK phosphorylation is influenced by CaMKK [50] and also change in the ATP/AMP ratio [51], it will be interesting to examine, in the future, whether CTXA affects expression and activity of CaMKK and alters the ATP/AMP ratio in 3T3-L1 preadipocyte differentiation process.

It is documented that lipolysis is a biological process in which ester bonds in TG are cleaved and generate free fatty acids and glycerol. Lipolysis is regarded as an alternative anti-obesity treatment [52]. Thus, any compound that enhances lipolysis can be used as potential anti-obesity therapeutics. In line with this, we herein have further tested the effect of CTXA on lipolysis in differentiated 3T3-L1 adipocytes. In the current study, we have observed that while 3 h treatment with ISO (20 μM), a lipolytic agent, leads to a big increase in glycerol release in differentiated 3T3-L1 adipocytes, CTXA at 5 to 20 μM does not induce glycerol release in these cells. HSL is a pivotal enzyme involved in lipolysis and its phosphorylation on several residues including S563 is indicative of an active form [53]. In this study, we have shown that while 3 h treatment with ISO (20 μM) induces high HSL phosphorylation on S563, that with CTXA at 5 to 20 μM does not. Taken together, these results point out that CTXA has no lipolytic effect on mature 3T3-L1 adipocytes. 

Obesity is alternatively defined as a chronic inflammatory disease [54]. It is known that (pre)adipocytes, a predominant cell type present in the adipose tissues, express and secrete not only adipokines (leptin, adiponectin, etc.) but also inflammatory cytokines (TNF-α, IL-1 β, IL-6, etc.) and enzymes (iNOS, COX-2, etc.) [21], which contribute to obesity inflammation. Studies have previously reported the ability of TNF-α to highly induce expression of COX-2 and iNOS in 3T3-L1 (pre)adipocytes [55]. However, until now, CTXA regulation of TNF-α-induced expression of COX-2 and iNOS in 3T3-L1 preadipocytes is not reported. In the present study, of interest CTXA at 10 µM for 4 h is able to largely interfere with TNF-α-induced expression of iNOS, but not COX-2, at both protein and mRNA level in 3T3-L1 preadipocytes, pointing out the specificity. These results thus advocate CTXA’s anti-inflammatory effect on preadipocytes, which may further contribute to its anti-obesity effect.

It should be noted that CTXA’s inhibitory effects on adipogenesis and inflammatory reaction herein is seen in cultured 3T3-L1 cells. Although not directly related to the current study, we have previously reported that CTXA protects sepsis-triggered renal damage in mice by inhibiting induction of iNOS expression [56] and attenuates sepsis-induced liver injury partially through the reduced expression of inflammatory cytokines including TNF-α [57], supporting this natural compound’s anti-inflammatory effect via iNOS and TNF-α down-regulation in vivo. It is known that inflammation contributes to the expansion of white adipocyte tissues by increasing adipogenesis, though the primary event triggering this remains unclear [58]. Overexpression and hyper-activation of inflammatory mediators in white adipose tissues partly leads to the development of obesity. Given that CTXA strongly interferes with TNF-α-induced iNOS expression in 3T3-L1 preadipocytes herein, it is conceivable that CTXA may inhibit adipogenesis by alleviating or resolving the TNF-α and iNOS-mediated inflammation in (pre)adipocytes in white adipose tissues. At this moment, it is not sure whether CTXA exerts its anti-adipogenic and anti-inflammatory effects in vivo. Future studies are warranted to evaluate if CTXA could inhibit adipogenesis in high-fat diet induced obese or Ob/Ob mice and its lipid-lowering effect is further linked with reduction of inflammation.

In summary, this is the first reporting that CTXA has strong anti-adipogenic and anti-inflammatory effects on 3T3-L1 cells, which are mediated through regulation of the expression and phosphorylation levels of C/EBP-α, PPAR-γ, STAT-3/5, FAS, ACC, perilipin A, AMPK, and iNOS. The present findings suggest that CTXA may be used as a potential anti-obesity natural substance.

## 4. Materials and Methods

### 4.1. Materials

Cudratricusxanthone A (CTXA) was isolated from the roots of *Cudrania tricuspidata* Bureau as reported previously [26]. Enhanced chemiluminescence (ECL) reagent was bought from Advansta (Menlo Park, CA, USA). 3-isobutyl-1-methylxanthine (IBMX), dexamethasone, and insulin were purchased from Sigma (St. Louis, MO, USA). Adipo-red assay reagent kit was bought from Lonza (Basel, Switzerland). Free Glycerol Reagent and Oil Red O working solution was obtained from Sigma (St. Louis, MO, USA). Pierce BCA Protein Assay Kit was bought from Thermo Scientific (Rockford, IL, USA). Antibodies used in this study are listed in Table S1.

### 4.2. Differentiation of 3T3-L1 Preadipocytes

3T3-L1 murine white preadipocytes (ATCC, Manassas, VA, USA) were grown up to the contact inhibition stage and remained in the post-confluent stage for 2 days in DMEM supplemented with 10% calf bovine serum (Gibco, Gaithersburg, MD, USA) and penicillin-streptomycin (Welgene, Daegu, Korea). Differentiation was induced by changing the medium to DMEM supplemented with 10% FBS (Welgene, Daegu, Korea) plus a cocktail of hormones (MDI) that include 0.5 mM IBMX (M) (Sigma, St. Louis, MO, USA), 0.5 µM dexamethasone (D) (Sigma), and 5 µg/mL insulin (I) (Sigma, St. Louis, MO, USA) in the absence or presence of CTXA at the indicated concentrations. In this study, the cell culture medium containing CTXA was vigorously vortexed for 30 s before addition to cells. After 48 h MDI-induction, the differentiation medium was replaced with DMEM supplemented with 10% FBS and 5 µg/mL I in the absence or presence of CTXA at the designated doses for additional 3 days. The cells were then fed every other day with DMEM containing 10% FBS in the absence or presence of CTXA at the indicated concentrations for additional 3 days.

### 4.3. Oil Red O Staining

On day 8 of differentiation, control or CTXA-treated 3T3-L1 cells were washed twice with PBS, fixed with 10% formaldehyde for 2 h at room temperature (RT), washed with 60% isopropanol, and dried completely. The fixed cells were then stained with Oil Red O working solution for 1 h at RT in the dark place and then washed twice with distilled water. Lipid droplets (LDs) accumulated in control or CTXA-treated 3T3-L1 cells were observed by light microscopy (Nikon, TS100, Tokyo, Japan).

### 4.4. Cell Count Analysis

3T3-L1 preadipocytes were seeded in 24-well plates. Cells were similarly grown under the above-mentioned differentiation conditions. On day 8 of differentiation, control or CTXA-treated 3T3-L1 cells, which cannot be stained with trypan blue dye, were counted under the microscope. The cell count assay was done in triplicates. Data are mean ± standard error (SE) of three independent experiments.

### 4.5. Quantification of Cellular TG Content 

Cells were similarly grown under the above-mentioned differentiation conditions. On day 8 of differentiation, intracellular TG content in control or CTXA-treated 3T3-L1 cells was measured using a commercially available Adipo-red assay reagent kit according to the manufacturer’s instructions (Lonza, Basel, Switzerland). Fluorescence was measured on Victor3 (Perkin Elmer, Waltham, MA, USA) with an excitation at 485 nm and emission at 572 nm. Data are mean ± SE of three independent experiments.

### 4.6. Preparation of Whole-Cell Lysates

At the designated time point, 3T3-L1 cells were washed twice with phosphate-buffered saline (PBS) and exposed to a modified radioimmunoprecipitation assay (RIPA) buffer (50 mM Tris-Cl (pH 7.4), 150 mM NaCl, 0.1% sodium dodecyl sulfate, 0.25% sodium deoxycholate, 1% Triton X-100, 1% Nonidet *p*-40, 1 mM EDTA, 1 mM EGTA, proteinase inhibitor cocktail (1×)). The cell lysates were then collected and centrifuged at 13,000 rpm for 15 min at 4 °C. The supernatant was saved, and protein concentrations were measured with Pierce BCA Protein Assay Kit (Thermo Scientific, Waltham, MA, USA). 

### 4.7. Western Blot Analysis

Proteins (30 µg) were separated by SDS-PAGE (10%) and transferred onto polyvinylidene difluoride (PVDF) membranes (Millipore, Billerica, MA, USA). The membranes were washed with Tri-buffered saline (TBS) (10 mM Tris, 150 mM NaCl) supplemented with 0.05% (*v*/*v*) Tween 20 (TBST) followed by blocking with TBST containing 5% (*w*/*v*) non-fat dried milk. The membranes were incubated overnight with specific primary antibodies listed in Table S1 at 4 °C. The membranes were washed three times with TBST at RT and then exposed to secondary antibodies coupled to horseradish peroxidase for 2 h at RT. The membranes were washed three times with TBST at RT. Immunoreactivities were detected by ECL reagents (Advansta, San Jose, CA, USA). Equal protein loading was assessed by the expression level of actin protein.

### 4.8. Quantitative Real-Time RT-PCR

Total cellular RNA in control or CTXA-treated 3T3-L1 cells was isolated with the RNAiso Plus (TaKaRa, Kusatsu, Shiga, Japan). Three µg of total RNA was used to prepare complementary DNA (cDNA) using a random hexadeoxynucleotide primer and reverse transcriptase. SYBR green (TaKaRa, Kusatsu, Shiga, Japan) was used to quantitatively determine transcript levels of genes with LightCycler96 Machine (Roche, Mannheim, Germany). PCR reactions were run in triplicate for each sample, and transcript levels of each gene were normalized to the expression level of 18S rRNA. Primer sequences used in this study are listed in Table S2. Data are mean ± SE of three independent experiments.

### 4.9. Reverse-Transcription Polymerase Chain Reaction (RT-PCR)

Three µg of total RNA were transcribed, the same method as in Section 4.8. The above-prepared cDNA was amplified by PCR with the primers listed in Table S3. The expression level of actin mRNA was used as an internal control as well as loading control. 

### 4.10. Measurement of Glycerol Content

Differentiated 3T3-L1 adipocytes (D8) were serum-starved for 2 h and incubated with different concentrations (5, 10, and 20 µM) of CTXA or isoproterenol (ISO, 20 µM), a known lipolysis inducer, for additional 3 h. The culture medium was saved, and glycerol content was measured by a free glycerol reagent according to the manufacturer’s instructions (Sigma). Absorbance was measured at a wavelength of 540 nm using the microplate reader. The assay was done in triplicates. Data are mean ± SE of three independent experiments.

### 4.11. Statistical Analyses

Cell count analysis was performed in triplicate and repeated three times. Data are expressed as mean ± SE. Statistical analysis was performed using SPSS v.11.5 software (SPSS, Inc. Chicago, IL, USA). Data were subjected to one-way ANOVA and Student *t*-test, followed by Dunnett’s post hoc test. *p* < 0.05 was considered to indicate statistically significant differences.

## Data Availability

Data is contained within the article.

## Supplementary Materials

The following are available online at https://www.mdpi.com/1422-0067/22/2/505/s1, Table S1: List of antibodies used for Western blot analysis. Table S2: Sequences of primers used for quantitative real-time PCR. Table S3: Sequences of primers used for Reverse-transcription polymerase chain reaction (RT-PCR).

## References

[1] Bluher M. (2019). Obesity: Global epidemiology and pathogenesis. Nat. Rev. Endocrinol..

[2] Sudhakaran M., Doseff A.I. (2020). The Targeted Impact of Flavones on Obesity-Induced Inflammation and the Potential Synergistic Role in Cancer and the Gut Microbiota. Molecules.

[3] Hill J.O., Wyatt H.R., Peters J.C. (2012). Energy balance and obesity. Circulation.

[4] Ali A.T., Hochfeld W.E., Myburgh R., Pepper M.S. (2013). Adipocyte and adipogenesis. Eur. J. Cell. Biol..

[5] Reilly S.M., Saltiel A.R. (2017). Adapting to obesity with adipose tissue inflammation. Nat. Rev. Endocrinol..

[6] Ghaben A.L., Scherer P.E. (2019). Adipogenesis and metabolic health. Nat. Rev. Mol. Cell. Biol..

[7] Onal G., Kutlu O., Gozuacik D., Dokmeci Emre S. (2017). Lipid Droplets in Health and Disease. Lipids Health Dis..

[8] Rosen E.D., MacDougald O.A. (2006). Adipocyte differentiation from the inside out. Nat. Rev. Mol. Cell Biol..

[9] Farmer S.R. (2006). Transcriptional control of adipocyte formation. Cell. Metab..

[10] Burrell J.A., Boudreau A., Stephens J.M. (2020). Latest advances in STAT signaling and function in adipocytes. Clin. Sci. (Lond.).

[11] Berndt J., Kovacs P., Ruschke K., Klöting N., Fasshauer M., Schön M.R., Körner A., Stumvoll M., Blüher M. (2007). Fatty acid synthase gene expression in human adipose tissue: Association with obesity and type 2 diabetes. Diabetologia.

[12] Cordonier E.L., Jarecke S.K., Hollinger F.E., Zempleni J. (2016). Inhibition of acetyl-CoA carboxylases by soraphen A prevents lipid accumulation and adipocyte differentiation in 3T3-L1 cells. Eur. J. Pharmacol..

[13] Kern P.A., Di Gregorio G., Lu T., Rassouli N., Ranganathan G. (2004). Perilipin expression in human adipose tissue is elevated with obesity. J. Clin. Endocrinol. Metab..

[14] Lage R., Diéguez C., Vidal-Puig A., López M. (2008). AMPK: A metabolic gauge regulating whole-body energy homeostasis. Trends Mol. Med..

[15] Martini C.N., Plaza M.V., Vila Mdel C. (2009). PKA-dependent and independent cAMP signaling in 3T3-L1 fibroblasts differentiation. Mol. Cell. Endocrinol..

[16] Prusty D., Park B.H., Davis K.E., Farmer S.R. (2002). Activation of MEK/ERK signaling promotes adipogenesis by enhancing peroxisome proliferator-activated receptor gamma (PPARgamma) and C/EBPalpha gene expression during the differentiation of 3T3-L1 preadipocytes. J. Biol. Chem..

[17] Engelman J.A., Lisanti M.P., Scherer P.E. (1998). Specific inhibitors of p38 mitogen-activated protein kinase block 3T3-L1 adipogenesis. J. Biol. Chem..

[18] Cani P.D., Amar J., Iglesias M.A., Poggi M., Knauf C., Bastelica D., Neyrinck A.M., Fava F., Tuohy K.M., Chabo C. (2007). Metabolic endotoxemia initiates obesity and insulin resistance. Diabetes.

[19] Cani P.D., Jordan B.F. (2018). Gut microbiota-mediated inflammation in obesity: A link with gastrointestinal cancer. Nat. Rev. Gastroenterol. Hepatol..

[20] Chirumbolo S., Franceschetti G., Zoico E., Bambace C., Cominacini L., Zamboni M. (2014). LPS response pattern of inflammatory adipokines in an in vitro 3T3-L1 murine adipocyte model. Inflamm. Res..

[21] Makki K., Froguel P., Wolowczuk I. (2013). Adipose Tissue in Obesity-Related Inflammation and Insulin Resistance: Cells, Cytokines, and Chemokines. ISRN Inflamm..

[22] Bai Y., Sun Q. (2015). Macrophage recruitment in obese adipose tissue. Obes. Rev..

[23] Ricciotti E., FitzGerald G.A. (2011). Prostaglandins and inflammation. Arterioscler. Thromb. Vasc. Biol..

[24] Förstermann U., Sessa W.C. (2012). Nitric oxide synthases: Regulation and function. Eur. Heart J..

[25] Kim H.L., Ha A.W., Kim W.K. (2020). Effect of saccharin on inflammation in 3T3-L1 adipocytes and the related mechanism. Nutr. Res. Pract..

[26] Kim T.J., Han H.J., Hong S.S., Hwang J.H., Hwang B.Y., Yoo H.S., Jin Y.R., Lee J.J., Yu J.Y., Lee K.H. (2007). Cudratricusxanthone A isolated from the root bark of Cudrania tricuspidata inhibits the proliferation of vascular smooth muscle cells through the suppression of PDGF-receptor beta tyrosine kinase. Biol. Pharm. Bull..

[27] Jeong G.S., Lee D.S., Kim Y.C. (2009). Cudratricusxanthone A from Cudrania tricuspidata suppresses pro-inflammatory mediators through expression of anti-inflammatory heme oxygenase-1 in RAW264.7 macrophages. Int. Immunopharmacol..

[28] Jeon S.M., Lee D.S., Jeomg G.S. (2016). Cudraticusxanthone A isolated from the roots of Cudrania tricuspidata inhibits metastasis and induces apoptosis in breast cancer cells. J. Ethnopharmacol..

[29] Choi E.H., Kim E.N., Jeong G.S. (2018). Inhibitory effect of Cudratricusxanthone A on osteoclast differentiation and function. Phytomedicine.

[30] Yoon C.S., Kim D.C., Quang T.H., Seo J., Kang D.G., Lee H.S., Oh H., Kim Y.C. (2016). A Prenylated Xanthone, Cudratricusxanthone A, Isolated from Cudrania tricuspidata Inhibits Lipopolysaccharide-Induced Neuroinflammation through Inhibition of NF-κB and p38 MAPK Pathways in BV2 Microglia. Molecules.

[31] Cohen P., Spiegelman B.M. (2015). Brown and Beige Fat: Molecular Parts of a Thermogenic Machine. Diabetes.

[32] Smith S., Witkowski A., Joshi A.K. (2003). Structural and functional organization of the animal fatty acid synthase. Prog. Lipid Res..

[33] Itabe H., Yamaguchi T.N., Nimura S., Sasabe N. (2017). Perilipins: A diversity of intracellular lipid droplet proteins. Lipids Health Dis..

[34] Oakhill J.S., Scott J.W., Kemp B.E. (2009). Structure and function of AMP-activated protein kinase. Acta. Physiol. (Oxf.).

[35] Winder W.W., Hardue D.G. (1999). AMP-activated protein kinase, a metabolic master switch: Possible roles in type 2 diabetes. Am. J. Physiol..

[36] Ahmad B., Serpell C.J., Fong I.L., Wong E.H. (2020). Molecular mechanism of adipogenesis: The anti-adipogenic role of AMP activated protein kinase. Front. Mol. Biosci..

[37] Wu L., Whang L., Li B., Jiang H., Duan Y., Xie Z., Shuai L., Li J., Li J. (2018). AMP-Activated Protein Kinase(AMPK) Regulates Energy Metabolism through Modulating Thermogenesis in Adipose Tissue. Front. Physiol..

[38] Jeon S.M. (2016). Regulation and function of AMPK in physiology and diseases. Exp. Mol. Med..

[39] Daval M., Foufelle F., Ferré P. (2006). Functions of AMP-activated protein kinase in adipose tissue. J. Physiol..

[40] Park Y.K., Obiang-Obounou B.W., Lee K.B., Choi J.S., Jang B.C. (2018). AZD1208, a pan-Pim kinase inhibitor, inhibits adipogenesis and induces lipolysis in 3T3-L1 adipocytes. J. Cell. Mol. Med..

[41] Lapid K., Graff J.M. (2017). Form(ul)ation of adipocytes by lipids. Adipocyte.

[42] Cao Z., Umek R.M., McKnight S.L. (1991). Regulated expression of three C/EBP isoforms during adipose conversion of 3T3-L1 cells. Genes Dev..

[43] Lefterova M.I., Lazar M.A. (2009). New developments in adipogenesis. Trends Endocrinol. Metab..

[44] Lehrke M., Lazar M.A. (2005). The many faces of PPAR gamma. Cell.

[45] Londos C., Brasaemle D.L., Schultz C.J., Segrest J.P., Kimmel A.R. (1999). Perilipins, ADRP, and other proteins that associate with intracellular neutral lipid droplets in animal cells. Semin. Cell. Dev. Biol..

[46] Wolins N.E., Brasaemle D.L., Bickel P.E. (2006). A proposed model of fat packaging by exchangeable lipid droplet proteins. FEBS Lett..

[47] Chen S., Li Z., Li W., Shan Z., Zhu W. (2011). Resveratrol inhibits cell differentiation in 3T3-L1 adipocytes via activation of AMPK. Can. J. Physiol. Pharmacol..

[48] Lee H., Kang R., Bae S., Yoon Y. (2011). AICAR, an activator of AMPK, inhibits adipogenesis via the WNT/β-catenin pathway in 3T3-L1 adipocytes. Int. J. Mol. Med..

[49] Poudel B., Lim S.W., Ki H.H., Nepali S., Lee Y.M., Kim D.K. (2014). Dioscin inhibits adipogenesis through the AMPK/MAPK pathway in 3T3-L1 cells and modulates fat accumulation in obese mice. Int. J. Mol. Med..

[50] Gormand A., Henriksson E., Strom K., Jensen T.E., Sakamoto K., Goransson O. (2011). Regulation of AMP-activated protein kinase by LKB1 and CaMKK in adipocytes. J. Cell. Biochem..

[51] Gowans G.J., Hardie D.G. (2014). AMPK: A cellular energy sensor primarily regulated by AMP. Biochem. Soc. Trans..

[52] Duncan R.E., Ahmadian M., Jaworski K., Sarkadi-Nagy E., Sul H.S. (2007). Regulation of lipolysis in adipocytes. Annu. Rev. Nutr..

[53] Anthonsen M.W., Rönnstrand L., Wernstedt C., Degerman E., Holm C. (1998). Identification of novel phosphorylation sites in hormone-sensitive lipase that are phosphorylated in response to isoproterenol and govern activation properties in vitro. J. Biol. Chem..

[54] Ellulu M.S., Patimah I., Khaza’ai H., Rahmat A., Abed Y. (2017). Obesity and inflammation: The linking mechanism and the complications. Arch. Med. Sci..

[55] Cheng A.W., Tan X., Sun J.Y., Gu C.M., Liu C., Guo X. (2019). Catechin attenuates TNF-α induced inflammatory response via AMPK-SIRT1 pathway in 3T3-L1 adipocytes. PLoS ONE.

[56] Lee W., Lee Y., Jeong G.S., Ku S.K., Bae J.S. (2017). Cudratricusxanthone A attenuates renal injury in septic mice. Food Chem. Toxicol..

[57] Lee Y., Jeong G.S., Kim K.M., Lee W., Bae J.S. (2018). Cudratricusxanthone A attenuates sepsis-induced liver injury via SIRT1 signaling. J. Cell. Physiol..

[58] Liu W., Li D., Cao H., Li H., Wang Y. (2020). Expansion and inflammation of white adipose tissue-focusing on adipocyte progenitors. Biol. Chem..

