# Supplementary material for: Cudratricusxanthone A Inhibits Lipid Accumulation and Expression of Inducible Nitric Oxide Synthase in 3T3-L1 Preadipocytes
Source: Int J Mol Sci. 2021 Jan 6;22(2):505. doi: 10.3390/ijms22020505 (PMC7825570; doi:10.3390/ijms22020505)
Supplement: Supplementary file 1 [file ijms-22-00505-s001.pdf]

Table S1: List of antibodies used for Western blot analysis.

Antibodies	Dilution used	Source	Catalog no.
Primary antibodies			
C/EBP-α	1:2,000	Santa Cruz Biotechnology	sc-61
PPAR-γ	1:2,000	Santa Cruz Biotechnology	sc-7273
p-STAT-3 (Y705)	1:2,000	Santa Cruz Biotechnology	sc-8059
STAT-3	1:2,000	Santa Cruz Biotechnology	sc-8019
p-STAT-5 (Y694)	1:2,000	Santa Cruz Biotechnology	sc-101806
STAT-5	1:2,000	Santa Cruz Biotechnology	sc-835
iNOS	1:2000	Santa Cruz Biotechnology	sc-651
Perilipin A	1:2,000	BioVision	#3948-200
FAS	1:2,000	BD Bioscience	#610962
p-AMPKα (T172)	1:2,000	Cell signalling	#2535
AMPK	1:2,000	Cell signalling	#2793
p-LKB1 (S428)	1:2,000	Cell signalling	#3482
LKB1	1:2,000	Cell signalling	#3047
p-ACC (S79)	1:2,000	Cell signalling	#3661
ACC	1:2,000	Cell signalling	#3662
p-HSL (S563)	1:2,000	Cell signalling	#4139
HSL	1:2,000	Cayman chemical	#10006371
COX-2	1:2000	Cayman chemical	#160106
β-Actin	1:10,000	Sigma	A5441
Secondary antibodies			
Goat anti-rabbit IgG-HRP	1:2000	Jackson ImmunoResearch Laboratories	111-035-045
Goat anti-mouse-IgG-HRP	1:2000	Jackson ImmunoResearch Laboratories	115-035-062

Table S2: Sequences of primers used for quantitative real-time PCR.

Gene	Forward	Reverse
C/EBP-α	TTACAACAGGCCAGGTTTCC	GGCTGGCGACATACAGTACA
PPAR-γ	AGGCCGAGAAGGAGAAGCTGTTG	TGGCCACCTCTTTGCTCTGCTC
FAS	TTGCTGGCACTACAGAATGC	AACAGCCTCAGAGCGACAAT
Perilipin A	CACTCTCTGGCCATGTGGA	AGAGGCTGCCAGGTTGTG
Leptin	GACCGGGAAAGAGTGACAGG	AGAGCAATCTGACACCAGCC
Adiponectin	ACGACACCAAAAGGGCTCAG	CGTCATCTTCGGCATGACTG
ACC	CAAGTGCTCAAGTTTGGCGC	CAAGAACCACCCCGAAGCTC
18S rRNA	GTAACCCGTTGAACCCCATT	CCATCCAATCGGTAGTAGCG

Table S3: Sequences of primers used for Reverse-transcription polymerase chain reaction (RT-PCR).

Gene	Forward	Reverse
COX-2	TTGAAGACCAGGAGTACAGC	GGTACAGTTCCATGACATCG
iNOS	GACAAGCTGCATGTGACATC	GCTGGTAGGTTCCTGTTGTT
Actin	TCATGAAGTGTGACGTTGACATCCGT	CCTAGAAGCATTTGCGGTGCACGATG
